# N-methylisatin-beta-thiosemicarbazone derivative (SCH 16) is an inhibitor of Japanese encephalitis virus infection *in vitro *and *in vivo*

**DOI:** 10.1186/1743-422X-5-64

**Published:** 2008-05-22

**Authors:** Liba Sebastian, Anita Desai, Madhusudana N Shampur, Yogeeswari Perumal, D Sriram, Ravi Vasanthapuram

**Affiliations:** 1 Department of Neurovirology, National Institute of Mental Health and Neuro Sciences, Bangalore-560029, India; 2 Department of Pharmacy, Birla Institute of Technology and Sciences, Pilani-333031, India

## Abstract

**Background:**

During the early and mid part of 20^th ^century, several reports described the therapeutic effects of N-methylisatin-β-Thiosemicarbazone (MIBT) against pox viruses, Maloney leukemia viruses and recently against HIV. However, their ability to inhibit flavivirus replication has not been investigated. Hence the present study was designed to evaluate the antiviral activity of 14 MIBT derivatives against Flaviviruses that are prevalent in India such as Japanese Encephalitis Virus (JEV), Dengue-2 (Den-2) and West Nile viruses (WNV).

**Results:**

Amongst the fourteen Mannich bases of MIBT derivatives tested one compound – SCH 16 was able to completely inhibit *in vitro *Japanese encephalitis virus (JEV) and West Nile virus (WNV) replication. However no antiviral activity of SCH 16 was noted against Den-2 virus replication. This compound was able to inhibit 50% of the plaques (IC_50_) produced by JEV and WNV at a concentration of 16 μgm/ml (0.000025 μM) and 4 μgm/ml (0.000006 μM) respectively. Furthermore, SCH 16 at a concentration of 500 mg/kg body weight administered by oral route twice daily was able to completely (100%) prevent mortality in mice challenged with 50LD_50 _JEV by the peripheral route. Our experiments to understand the mechanism of action suggest that SCH 16 inhibited JEV replication at the level of early protein translation.

**Conclusion:**

Only one of the 14 isatin derivatives -SCH 16 exhibited antiviral action on JEV and WNV virus infection *in vitro*. SCH 16 was also found to completely inhibit JEV replication *in vivo *in a mouse model challenged peripherally with 50LD_50 _of the virus. These results warrant further research and development on SCH 16 as a possible therapeutic agent.

## Background

Flaviviruses are considered to be important pathogens responsible for significant human morbidity and mortality. The World Health Organization estimated that more than 50 million Dengue viral infections and 50,000 cases of Japanese encephalitis occur annually worldwide [1]. Severe manifestations of flavivirus disease include hemorrhagic fever, encephalitis and neurological sequelae. Despite the major clinical and public health impact of flaviviruses, there are no drugs available for chemoprophylaxis or chemotherapy of these infections. The advent of potent combination antiretroviral therapy has been an important breakthrough in the treatment of HIV-1 infection, resulting in marked reductions in HIV-1-related morbidity and mortality [2]. This has rekindled interest in the search for antiviral agents for a variety of viral infections.

Earlier reports have described antiviral activity of some compounds against flaviviruses [3]. However, only a few of them have described both *in vitro *and *in vivo *activity of antiviral agents against flaviviruses [3]. Thiosemicarbazones were the first antiviral compounds recognized to have a broad-spectrum antiviral activity against a range of DNA and RNA viruses [4,5]. The use of N-methylisatin-β-thiosemicarbazone (methisazone/marboran) as an effective antiviral drug in the chemoprophylaxis of small pox was demonstrated in human volunteers in South India as early as 1965 [6]. In several trials during Indian epidemics methizasone proved its value by reducing the attack rates by 75 to 95% [6]. Similarly, other studies have shown that Methyl isatin-β-diethylthiosemicarbazone inhibits replication of Moloney Leukemia Virus by interfering with the early phase of viral life cycle [7]. However, the antiviral activity of isatin thiosemicarbazone derivatives has not been evaluated against flaviviruses. Therefore, this study was undertaken to investigate if any of the N-methylisatin-β-thiosemicarbazone derivatives could suppress common flavivirus infections encountered in South India such as Japanese Encephalitis, Dengue and West Nile viral infections. The aim was not to develop a clinical protocol for therapy of these infections but rather to investigate the possibility of identifying antiviral agents that could target flavivirus multiplication.

## Results

### Antiviral screening of compounds *in vitro *by cytopathic inhibition assay

**Figure 1:**
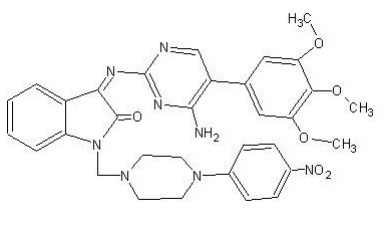
Butterfly structure of N-Methylisatin-β-Thiosemicarbazone derivative SCH 16.

**Figure 2:**
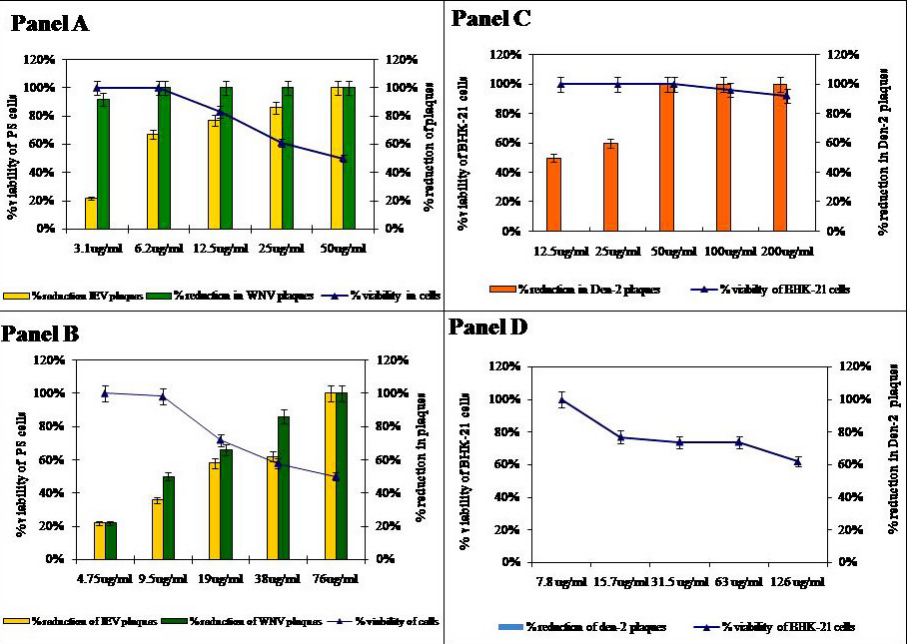
**Antiviral activity of Ribavirin and SCH 16 against JEV, WNV and Den-2 evaluated using the plaque reduction assay**. ***Panel A***: Represents the dose dependent reduction in JEV (yellow bars) and WNV (green bars) plaques obtained in PS cells with the standard antiviral agent Ribavirin (represented as bars). The X axis represents the various concentrations of the compound, Y' axis represents the percent reduction in plaques. The viability of cells is represented as line graph superimposed on the bar diagram on the Y axis.   ***Panel B***: Represents the dose dependent reduction in JEV (yellow bars) and WNV (green bars) plaques obtained in PS cells with the SCH 16 (represented as bars). The X axis represents the various concentrations of the compound, Y' axis represents the percent reduction in plaques. The viability of cells is represented as line graph superimposed on the bar diagram on the Y axis.   ***Panel C***: Represents the dose dependent reduction in Den-2 plaques (orange bars) obtained in BHK 21 cells with the Ribavirin (represented as bars). The X axis represents the various concentrations of the compound, Y' axis represents the percent reduction in plaques. The viability of cells is represented as line graph superimposed on the bar diagram on the Y axis.   ***Panel D***: Note that there was no reduction of in Den-2 plaques was obtained with the SCH 16 in BHK 21 cells. The X axis represents the various concentrations of the compound, Y' axis represents the percent reduction in plaques. The viability of cells is represented as line graph superimposed on the bar diagram on the Y axis.

Initially, the 50% Cytotoxic Concentration (CC_50_) of the 14 MIBT derivatives and Ribavirin were determined on Porcine Stable kidney (PS) and Baby hamster kidney (BHK 21) cell lines and the results are depicted in Table 1. The antiviral activity of the 14 MIBT derivatives were initially evaluated against JEV, WNV and Den-2 using Cytopathic Effect (CPE) inhibition assay and it was observed that only SCH 16 showed inhibition of CPE. The structure of this MIBT derivative is depicted in Figure 1. Ribavarin, a known inhibitor of flavivirus was used as a control in all the experiments. Although there is no structural similarity between Ribavarin and SCH 16, we opted to use Ribavarin as a positive control in all experiments so that we have a reference value for comparing the results of SCH 16. These two compounds were then subjected to evaluation by the plaque reduction assay at non-cytotoxic concentrations (<CC_50_). It was noted that SCH 16 and Ribavirin exhibited a dose depended reduction of plaques formed by JEV and WNV (Figure 2, Panels A and B) with an IC_50 _of 16 μg/ml (0.000025 μM) and 4 ug/ml (0.000006 μM) for JEV and WNV respectively. On the contrary the IC_50 _of Ribavirin was 3.9 μg/ml (0.000016 μM) and 1.7 μg/ml (0.000007 μM) for JEV and WNV respectively. No antiviral activity of SCH 16 was noted against Den-2 although Ribavarin showed a dose dependent inhibition of Den-2 plaque formation (Figure 2, Panels C and D).

**Table 1:** List of Methylisatin-β-thiosemicarbazone (MIBT) derivatives and the CC_50 _on PS and BHK-21 cells

Compounds	*CC_50 _on PS cell line	*CC_50 _on BHK-21 cell line
1 SF3	47 μg/ml	86 ug/ml
2 SF7	43 μg/ml	200 ug/ml
3 SCH16	76 μg/ml	126 ug/ml
4 SF17	21 μg/ml	42 ug/ml
5 SCH17	41 μg/ml	46 ug/ml
6 SC18	17 μg/ml	82 ug/ml
7 SCH19	18 μg/ml	141 ug/ml
8 SC18	31.5 μg/ml	140 ug/ml
9 SB18	22.5 ug/ml	36 ug/ml
10 SF24	25.5 ug/ml	16.8 ug/ml
11 SF27	22.5 ug/ml	51 ug/ml
12 SC27	21 ug/ml	46 ug/ml
13 SC28	21 ug/ml	94 ug/ml
14 SB29	25 ug/ml	21.5 ug/ml
15.Ribavirin	50 ug/ml	200 ug/ml

*CC_50 _= The concentration of the compound that reduced the viability of cells to 50% of the control. Note: The 14 MIBT compounds belonged to four different categories based on the halogen or methyl group substituted at the position R and are designated as SB group with bromine, SC group with chlorine, SF group with fluorine and SCH group with -CH3 group substituted at R. R' has N-substituted aromatic side chain attached to the -CH2 moiety. Cytotoxicity concentration (CC_50_) of synthesized compounds was evaluated on exponentially growing PS and BHK-21 cells. It can be observed that the CC_50 _of MIBT derivatives ranged from ≥ 76 ug/ml to ≥ 17 ug/ml, while CC_50 _on BHK-21 cell line ranged from ≥ 200 ug/ml to ≥ 16.8 ug/ml.

The specificity of the action of an antiviral compound is determined by calculating the Therapeutic Index (TI), which is the ratio of CC_50 _to IC_50_. The TI of SCH 16 was 5 and 19 for JEV and WNV respectively while for Ribavirin it was 13 and 29 respectively. This suggests that SCH 16 is moderately active against JEV and highly active against WNV.

### The kinetics of action of SCH 16 in relation to the replicative cycle of JEV *in vitro*

As a first step to understand JEV and SCH 16 interactions, experiments were designed to determine the kinetics of JEV replication *in vitro*. It was noted that the earliest appearance of JEV antigen in infected PS cells was at 10 hours post-adsorption as detected by IFA (data not presented). However, the first infectious progeny of virus was detected in the supernatant medium at 14 hours post-adsorption thereby suggesting that a single replicative cycle of JEV *in vitro *in PS cell line requires 14 hours for completion (data not presented).

**Figure 3:**
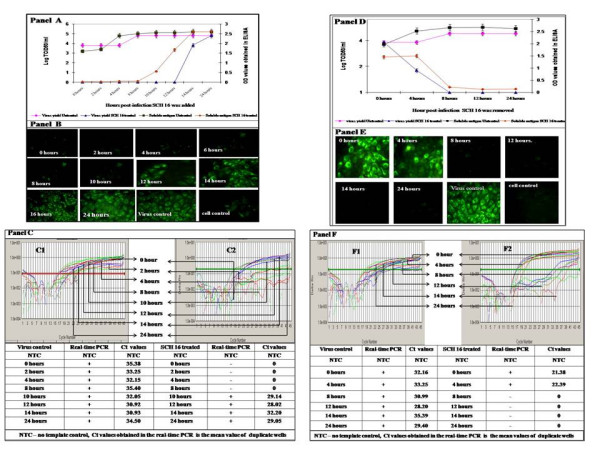
**Kinetics of action of SCH 16 in relation to the replicative cycle of JEV in PS cells.*Panel ****A *represents the results of the experiments wherein the addition of the drug SCH 16 to virus infected PS cells was staggered (refer to Material & Methods for details). X axis represents the various time points at which SCH 16 was added after adsorption of JEV onto PS cells. Note that there was no virus yield (represented as Log TCID_50_/ml on Y axis) in drug treated cells (blue triangle) until 8 hours post infection after which virus yield steadily increased to attain levels similar to that obtained in untreated cells (pink sphere). The Y' axis represents the optical density values obtained in the JEV antigen capture ELISA. Soluble JEV antigen was measured in the supernatant fluids obtained at 48 hrs after the experiment (refer to Material & Methods for details) in both drug treated (black square) and untreated (red diamond) cells. ***Panel B ***depicts the detection of JEV specific antigen using an immunofluorescent assay. Note the presence of bright immunofluorescence in the JEV infected monolayers (virus control). It can also be observed that JEV infected mono layers treated with SCH 16 were positive for viral antigen at 10, 12 and 14 hours post infection whilst viral antigen was undetectable by immunofluorescence at 0, 2, 4, 6 and 8 hrs post infection respectively (400×). ***Panel C***: The amplification plots obtained in Real Time PCR depicting the detection of JEV RNA in the untreated cells and SCH 16 treated cells at varying time points post-infection. Panel ***C-1 ***depicts the typical amplification plot (fluorescence vs cycle number) obtained by the real time PCR with the RNA extracted from the virus infected untreated cells at varying time points. Note that JEV RNA was detected at all time points. In contrast JEV RNA was undetectable at 0, 2,4, and 8 hrs in the SCH 16 treated cells (Panel C2). ***Panel D ***represents the results of the experiments wherein the minimum time required for SCH 16 to exert antiviral activity was evaluated (refer to Materials & Methods for details). SCH 16 was added to all monolayers 2 hrs post virus adsorption and removed from the mono layers at periodic intervals. X-axis represents the various time points when SCH 16 was removed after JEV entry into PS cells. Note that virus yield (represented as Log TCID_50_/ml on Y axis) in drug treated cells (blue triangle) steadily declined from 0 hrs post infection until 8 hours post infection after which there was no virus production noted in drug treated cells. On the contrary virus yields continued to be high in untreated cells (pink sphere) at all time points. The Y' axis represents the optical density values obtained in the JEV antigen capture ELISA. Soluble JEV antigen was measured in the supernatant fluids obtained at 48 hrs after infection (refer to Materials & Methods for details) in both drug treated (black square) and untreated (red diamond) cells. ***Panel E***: Effect of duration of antiviral action of SCH 16 on JEV replication post-infection. SCH 16 was added to JEV infected PS cell monolayer at 0 hours post – adsorption and the inoculums were removed at different time points post-infection (0 to 14 hrs). The monolayer was stained using JEV specific monoclonal antibodies by IFA at 48 hours (400×). Presence of cell bound antigen can be appreciated upon the removal of SCH 16 in the early hours (up to 4 hours) of viral replicative cycle, while viral antigen was not detected when SCH 16 was retained with the infected monolayer for longer duration (8 hours andmore). ***Panel F***: The amplification plots obtained in Real Time PCR depicting the detection of JEV RNA in the infected cells treated with SCH 16 at 0 hours and inoculums removed at varying time points (refer to Materials and methods for details). Panel F***-1 ***depicts the typical amplification plot (fluorescence vs cycle number) obtained by the real time PCR with the RNA extracted from the virus infected untreated cells at varying time points. Note that JEV RNA was detected at all time points. In contrast JEV RNA was detectable only at 0 and 4 hrs in the SCH 16 treated cells and undetectable beyond 8 hrs (Panel F2).

The antiviral activity of SCH 16 was subsequently investigated in relation to the kinetics of JEV replication. Non-toxic concentration of SCH 16 was added at various time points following entry of JEV into PS cells and the experiments terminated following 48 hours incubation. The compound at a concentration of 76 μg/ml (0.00012 μM) was able to completely inhibit JEV replication when added to the infected monolayer at 2, 4, 6 and 8 hours post-infection evidenced by the absence of viral RNA, viral antigen and inhibition of virus yield (Figure 3, Panel A to C). However, addition of SCH 16 beyond 8 hours post infection did not completely inhibit JEV replication since JEV antigen, RNA and infectious virus were detected at subsequent time points (Figure 3, Panels A to C).

In order to determine the minimum contact period required for SCH 16 to exert its antiviral effect on JEV replication *in vitro*, a series of experiments were performed. SCH 16 was added to JEV infected cell cultures at '0' hour post-infection and removed at 4 hourly time points up to 14 hours and the monolayers were further incubated for 48 hours at 37°C under 5% CO_2_. It was observed that there was complete inhibition of virus replication when SCH 16 was allowed to be in contact with infected cultures for more than 8 hours post-infection. However, when SCH16 was withdrawn at earlier time points there was no inhibition of virus replication as confirmed by the detection of viral antigen, viral RNA and infectious virus yield (Figure 3, Panels D to F).

### Effect of SCH 16 on viral translation

**Figure 4:**
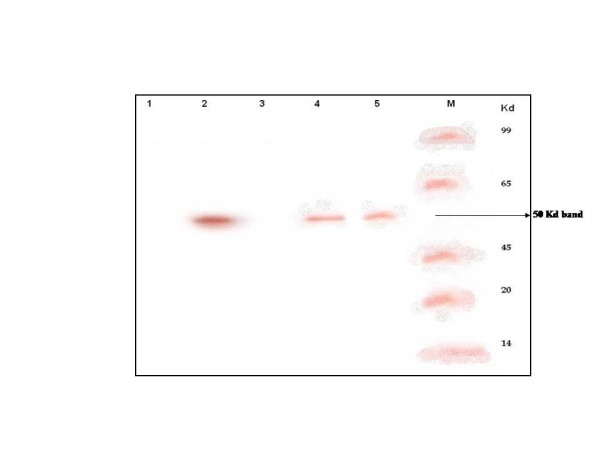
**Western blot illustrating the effect of SCH 16 on JEV translation using an *in vitro *translation kit.** Lane 1-uninfected cell control, lanes 2 and 4 – in vitro translation products of RNA obtained form JEV infected PS cells (untreated) at 4 and 10 hours post infection respectively. Lanes 3 and 5 – in *vitro *translation product of RNA obtained from JEV infected PS cells treated with SCH 16 for 4 hours and 10 hours respectively. Note that SCH 16 treatment of JEV infected cells did not show any in vitro translation product at 4 hours post treatment (Lane 3) whilst at 10 hours (Lane 5) a 50 Kda *in vitro *translation product was obtained. Lane M represents molecular weight markers.

To understand the probable action of SCH 16 on the viral replicative cycle and to study the extent of damage caused by the compound on the viral RNA that might result in the inhibition of viral events such as protein synthesis (translation), an *in vitro *translation experiment was carried out as described in materials and methods. RNA was extracted from drug treated (4 hours and 10 hours post infection) and untreated monolayers of JEV infected cells and subjected to Real Time PCR analysis to confirm the presence of JEV RNA. Subsequently, the viral RNA was subjected to *in vitro *translation. It was observed that RNA extracted from JEV infected cells treated with SCH 16 for 4 hours failed to translate into JEV proteins *in vitro*. On the contrary, viral RNA extracted from infected cells treated with SCH 16 at 10 hours as well as RNA from infected cells that were not treated with SCH 16 showed the presence of JEV proteins (Figure 4).

### In vivo evaluation of compounds against JEV using mouse model

After ascertaining the *in vivo *non-toxic concentrations in preliminary experiments, the therapeutic potential of SCH 16 was evaluated in mice using intracerebral and intraperitoneal challenge routes. In the intracerebral challenge model, mice that were treated with 100 and 200 mg/kg body weight of SCH 16 showed no protection. However, it was interesting to note that all the mice that were treated with SCH 16 remained healthy up to day 6 post-infection without showing any apparent symptoms of JEV infection (data not presented). The symptoms started appearing in these mice from day 7 post-infection. There was a gradual progression of the symptoms and death occurred on day 9. On the other hand, untreated mice appeared sick by day 3 and succumbed by day 5. This suggests that there was a prolonged survival time of 3 days between the treated and untreated mice.

**Figure 5:**
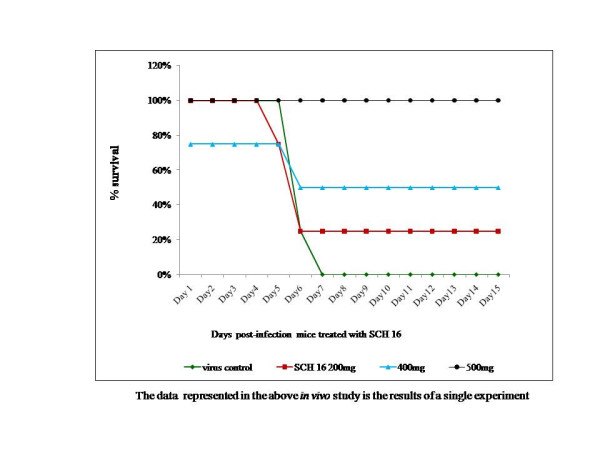
**Survival graphs depicting the *in vivo *effect of SCH 16 against a lethal JEV challenge. **SCH 16 was administered to swiss albino mice (n = 4 per group) per orally twice daily at 12 hour intervals to three groups of mice. Each group of mice received the drug at 200 (red square), 400 (blue triangle) and 500 (black sphere) mg/kg body weight respectively. A forth group of mice (n = 4) served as virus control (green diamond) and did not receive the drug. All the groups of mice were challenged with 50LD_50_JEV (P20778) by intraperitoneal route as described in materials and methods. The survival of mice was monitored for 20 days post-challenge. X-axis depicts the days post challenge. Y-axis depicts the percentage survival of mice treated with various concentrations of the drug as wells untreated control mice. Each data point depicts the mean survival rate of four mice in the respective group. Note that all mice in the virus control group succumbed by 7 days post challenge.

The prolonged survival time observed in the intracerebral challenge experiments prompted us to make use of a peripheral challenge model (JEV 50LD_50_) using a multiple dosage regimen wherein 200, 400 and 500 mg/kg body weight of SCH 16 was administered by oral route. It was observed that, there was 25% protection in the group of mice administered with 200 mg/kg body weight of SCH 16, 50% protection observed in the group that received 400 mg/kg body weight and complete protection was observed in the group that were given with 500 mg/kg body weight of SCH 16 (Figure 5). Mice that survived the challenge post treatment were sacrificed; brains harvested and subjected to virus isolation, detection of viral antigen and viral RNA. Viable virus could not be isolated from the brain tissue of these mice. Further, no viral antigen could be demonstrated in the brain smears by immunofluorescent staining using monoclonal antibodies to JEV. However, the RT-PCR products amplified from the brain homogenate suggested that viral RNA was present in the brain of animals that survived JEV infection following treatment with 400 and 500 mg/kg body weight of SCH 16.

## Discussion

There is currently no specific antiviral treatment available for Japanese encephalitis, West Nile and Dengue virus infections. Recently there has been renewed interest in the search for antiviral compounds active against a variety of viral infections. For instance, there are several reports describing the *in vitro *inhibitory effect of compounds such as ribavirin, mycophenolic acid, imino sugars, inhibitors of serine protease, RNA interference and non-steroidal anti-inflammatory drugs against flaviviruses [8-13]. N-Methylisatin-β-thiosemicarbazone (MIBT) was one of the first antiviral compounds to be discovered. It exhibits antiviral activity against a variety of RNA and DNA viruses [14-17]. Recent studies have demonstrated that thiosemicarbazone and Mannich bases of thiosemicarbazone derivatives exhibit anti-HIV activity *in vitro *[18-22]. Therefore this study was designed to investigate the antiviral property of isatin β thiosemicarbazone derivatives against JEV, WNV and Den-2 viruses.

In the present study, fourteen Mannich bases of MIBT derivatives were synthesized and evaluated for their ability to inhibit flaviviral replication. However, only one compound (SCH 16) showed antiviral activity against JEV and WNV *in vitro *with a therapeutic index of 5 and 16 respectively. This compound did not exhibit any virus inactivating property. SCH 16 (Figure 1) is a mannich base of N-Methylisatin-β-thiosemicarbazone possessing an isatin backbone with modifications made at the side chains. Chemically isatins are diketonic compounds. It has been earlier noted that, heteroaromatic thioamides containing N-substitution at more than one position per heterocyclic ring are worthy of investigation due to its increased antiviral property [4]. It is therefore likely that the antiviral activity of SCH 16 may be due to the N substitution at the 8th position in the heterocyclic benzene ring and a NO2 group attached to the aromatic side chain.

Although SCH16 exhibited antiviral activity against WNV, we did not pursue further experiments with it since WNV is not a public health concern in India. In contrast, JEV is a major public health problem in India and hence we set about to investigate in detail the mechanism of antiviral activity of SCH 16 against JEV. Two crucial questions pertaining to the antiviral activity of SCH16 against JEV were addressed; (i) how long after virus infection can addition of drug be delayed *in vitro *in order to achieve inhibition of virus replication? and (ii) what is the minimum time required for SCH16 to exert its antiviral activity?. For this purpose we used an experimental approach similar to that described earlier by Baginiski et al and Lammarre et al [23,24]. Our results showed that when the drug was added to infected cells at various time points post virus entry, neither viral antigen (Figure 3 Panel A & B) nor viral nucleic acid (Figure 3, Panel C) was detected up to 8 hours post infection. Beyond this time point however, viral antigen, nucleic acid and infectious virus was detectable in the cultures. Indeed viral antigen, viral RNA and virus yields were comparable to those obtained with untreated cells beyond the 8 hour time point thereby suggesting that SCH 16 did not inhibit normal cellular functions (Figure 3, Panels A to C). This suggests that the drug was not toxic to cells and did not inhibit the ability of cells to support virus replication at later time points. To ascertain the minimum time required for SCH16 to exert its antiviral activity, the compound was added at 2 hours post infection and removed at various time points post viral entry. The results revealed that, SCH 16 probably acted as an inhibitor of early protein synthesis. Had SCH 16 been an un-coating inhibitor or a polymerase inhibitor, the drug would have required a contact time of less than 4 hours to bring about its inhibitory effect. Similarly if it were a protease inhibitor the minimum contact period for SCH 16 to bring about inhibition of virus replication would have been greater than 8–10 hours. Since we observed that the minimum contact period of 8 hours was required for SCH16 to completely inhibit virus replication, it probably indicates that the drug is acting at the level of translation. Cooper et al [25] in an earlier study with vaccinia virus had demonstrated that the specific antiviral effect of MIBT was noted 6 hours post-infection thereby indicating inhibition of viral protein synthesis. In order to ascertain whether this was indeed also true for SCH 16 we adopted another approach to investigate the precise role of SCH 16 on translation events in JEV replication. We obtained RNA samples from the experiments that treated JEV infected monolayer's with SCH 16 for 4 hours and SCH 16 added at 10 hours post infection from infected cells treated with SCH 16 as well as cells that were untreated using identical extraction protocols. Subsequently we performed Real Time SYBR Green I PCR using JEV specific primers to confirm the presence of JEV RNA in samples obtained from both drug treated as well as untreated cells. The viral RNA thus obtained, was then subjected to *in vitro *translation experiments which clearly showed that there were no translation products obtained with RNA obtained from drug treated cells at 4 hours post infection (Figure 4, lane 3). On the contrary, RNA obtained from drug treated cells at 10 hours post infection (Figure 4, lane5) as well as RNA obtained from untreated cells at both 4 hours and 10 hours post infection (Figure 4, lanes 2 and 4). This result demonstrates that SCH16 is able to selectively suppress translation of JEV RNA at early time points in the life cycle. Similar observations have been made earlier by Ronen et al on other RNA virus [26] who investigated the inhibitory action of N-methyl isatin beta-diethylthiosemicarbazones on Moloney Leukemia virus replication.

The therapeutic potential of SCH 16 against JEV was evaluated *in vivo *in mice using the intracerebral and intraperitoneal challenge studies. The mice that were evaluated in the intracerebral challenge route did not show any protection although there was a delay in appearance of symptoms and death in drug treated mice. The lack of protection by this route may be due to (i) the direct introduction of large amount of infectious virus (50LD_50_) into the CNS which might have compromised the inhibitory action of SCH 16 and/or (ii) inability to achieve therapeutic concentrations of the drug in the brain either due to delay in the compound reaching the brain from the intraperitoneal compartment or poor penetration of the drug into the brain parenchyma. On the contrary, the drug treated mice challenged by the intraperitoneal route showed a dose dependent reduction in mortality, whilst all the untreated mice succumbed to the challenge with 50LD_50 _of JEV by day seven (Figure 5). Furthermore, neither viable virus nor viral antigen could be demonstrated in the brains of the mice that survived the challenge. However, viral RNA was detected by real-time RT-PCR in all the brain tissues. Since flavivirus RNA dependent RNA polymerases are active within three hours of viral entry this is not a surprise finding [27]. Because, SCH 16 is primarily an early translation inhibitor, it appears that this drug does not interfere with RNA polymerization resulting in accumulation of viral RNA in the brains of drug treated mice that survived the challenge. Alternatively, SCH 16 treatment could have curtailed JEV replication in the periphery resulting in a very small amount of JEV entering the brain. Consequently the virus was unable to establish a productive infection in the brain and the presence of viral RNA could be as a result of residual virus in brains of mice that survived the challenge. In an experimental rat model, with post-encephalitic Parkinsonism induced by JEV infections [28,29] it was observed that, administration of isatin improved the motor neuron activities significantly. Indeed, they attributed that the improvement in the motor weakness was probably due to the MAO inhibitory activity of isatin and suggested that isatin could possibly serve as a new therapeutic agent for Parkinsonism. However, these studies were not designed to address the antiviral action of isatin against JEV but aimed at investigating the neurotransmitter inhibitory effect. It may be argued therefore that the *in vivo *effect of SCH 16 against JEV noted in this study may also be attributed to the immunomodulating or neuroprotective property of SCH 16.

An intriguing observation in this study was the differential ability of SCH 16 to suppress JEV, WNV and Den-2 multiplication *in vitro*. It is difficult to hypothesize the differential antiviral property of SCH 16 noted against JEV and WNV in this study as they are structurally similar and we have used the same cell system (PS cells) for evaluating the drug. On the contrary, we used BHK 21 cells for assaying the antiviral activity of SCH 16 against Den-2 virus, which could have contributed to the lack of anti-Dengue activity of SCH 16. Protein synthesis consists of an intricate series of events requiring components that are too numerous to be encoded by viral genomes [30,31]. It has been observed that Den-2 and other flaviviruses, such as WNV, yellow fever, JEV, and Kunjin viruses, are presumed to undergo cap-dependent translation [32,33]. However, evidence exists that under certain conditions that inhibit cap dependent translation, Den-2 viruses can switch to more efficient cap independent translation. Further, mammalian cellular stress response and immune functions, such as the interferon antiviral response [34,35], may compel viral translation by one mechanism over the other. Since we used PS cells for evaluation of JEV and WNV and BHK 21 cells for Den-2 it is possible that the translation pathway adopted by Den-2 against SCH 16 may be due to the presence of certain BHK 21 cell specific factors. However, strong experimental evidence is needed to support this hypothesis and it would be interesting to investigate whether SCH 16 is indeed a cap dependent translation inhibitor.

## Conclusion

In conclusion, the findings of this study unequivocally demonstrate that SCH 16 has antiviral activity against JEV and WNV *in vitro*. Furthermore, SCH 16 was also found to completely inhibit JEV replication *in vivo *in a mouse model challenged peripherally with 50LD_50 _of the virus in a dose dependent manner. This necessitates further investigation into the pharmacokinetcis of the compound. Its moderate therapeutic index (TI = 5) may be a concern. However, further investigation on structure – activity relationships and appropriate modification in the aryl ring of the isatin moiety could provide more effective JEV-inhibitors with improved efficacy in future.

## Materials and methods

### Viruses

Standard strains of JEV (P20778), Den-2 virus (P23085) and WNV (G22886) were obtained from National Institute of Virology (NIV), Pune, India.

### Cells and animals

*Aedes albopictus *(C6/36) mosquito cell line and Porcine Stable kidney (PS) cells were maintained in Minimum Essential Medium (MEM) with 10% fetal calf serum while Baby Hamster Kidney (BHK-21) cells were maintained in Dulbecco's MEM with 10% fetal calf serum (NCCS, Pune, India). Random bred Swiss albino mice (4–5 week old) were obtained from Central Animal Research Facility, NIMHANS, Bangalore, India, and used for the *in vivo *evaluation. All animal experiments were conducted after obtaining permission from Institutional Animal Ethics Committee.

### N-Methylisatinisatin-β-Thiosemicarbazone (MIBT) derivatives

Fourteen mannich bases of isatin-β-thiosemicarbazone derivatives (Table 1) were obtained from Dr. Sriram, Birla Institute of Technology and Science (BITS), Pilani, India. The compounds were synthesized by Schiff reaction. N, N-diethyl thiosemicarbazide was condensed with isatin in the presence of glacial acetic acid to form 1H-indole-2, 3-dione -3-N, N-diethyl thiosemicarbazone (Schiff base). The N-Mannich bases were further condensed using acidic imino group along with formaldehyde and various secondary amines to obtain isatin thiosemicarbazone derivatives. Ribavirin, which is a known inhibitor of flavivirus replication, was obtained from commercial sources (Sigma, USA) and used as a control drug in this study.

### Cytotoxicity of Ribavirin and MIBT derivatives

Cytotoxicity of the antiviral compounds was evaluated using the Trypan blue exclusion assay [36]. Briefly, PS and or BHK-21 cells grown to semi-confluence in 24-well plates were exposed to different concentrations of the compounds for 4 days at 37°C. Following this, the cells were harvested by trypsinization and re-suspended in 0.5 ml of MEM containing 10% FCS. A 100 μl of the cell suspension was mixed with 50 μl of 2.5% Trypan blue and the number of viable cells was enumerated using a hemocytometer. The concentration of compound that reduced cell growth by 50% was estimated as the 50% cytotoxic concentration (CC_50_). The effect of the compounds on cellular proliferation was also studied. Briefly, the drug treated cells and untreated cells were seeded at a rate of 2 × 10^4 ^cells per well into 24-well plates and allowed to proliferate for 3 days in MEM, containing 10% FCS. The proliferations of cells were monitored every day microscopically by recording signs of toxicity such as altered morphology presence or absence of vacuoles and/or dead cells.

### Screening for inhibition of virus induced cytopathic effect *in vitro*

The antiviral activity assay of the Ribavarin and MIBT derivatives against JEV, Den-2 virus or WNV were screened *in vitro *using the cytopathic effect (CPE) inhibition assay carried out in a 96 well plate. Briefly, monolayers of PS and/or BHK-21 were inoculated with 100 μl of appropriate virus suspension containing 1 MOI of virus and adsorbed for two hours at 37°C. At the end of incubation period, the virus (JEV, Den-2 or WNV) was removed and the monolayers were rinsed with MEM to remove unbound virus. Doubling dilutions of different concentrations of Ribavirin and MIBT derivatives (beginning with CC_50_) were prepared in MEM, added to the monolayer (100 μl) and incubated at 37°C for 3 days under 5% CO_2_. The experiment was terminated when the virus control showed maximum CPE. The presence or absence of CPE was recorded microscopically every day and the plates were stained using crystal violet at the termination of experiment and compared with the untreated virus controls and drug controls. All the experiments were run in triplicates to ensure reproducibility.

### Confirmation of antiviral activity by plaque reduction assay

The compounds that showed inhibition of virus replication in the CPE inhibition assay were further evaluated using plaque-reduction assay. Briefly, PS (4 × 10^4 ^cells/well) cells were grown to a confluent monolayer in a 24 well plate and infected with 100 μl of virus suspension containing 1 MOI of JEV and incubation was carried out for 2 hours at 37°C. At the end of adsorption, monolayers were rinsed with sterile PBS and 100 μl MEM containing varying concentrations of the compounds were added. The monolayer was then overlaid with maintenance medium containing 0.2% molten agarose (Sigma-Aldrich, USA). Appropriate controls were included in each run of the assay. Incubation was carried out at 37°C for 3 days. At the end of incubation period monolayers were fixed in 10% formal saline, the agarose was gently removed and the cells were stained using 1% crystal violet. Two independent observers counted the plaques using a hand lens. All the experiments were run in triplicates. Percentage inhibitions of plaques were determined using the formula given below.

$$\%\;\text{Inhibition}=\frac{\text{Number of plaques in virus control-Number of plaques in drug treated}}{\text{Number of plaques in virus control}}\times 100$$

The antiviral activity was expressed as 50% inhibitory concentration (IC_50_) of the compound, which is the concentration of the compound required to inhibit viral plaques by 50% as compared to virus control. The therapeutic potential and specificity of action was determined by calculating the Therapeutic Index (TI), which is the ratio of CC_50 _to IC_50 _(CC_50_/IC_50_) [37].

### Understanding the mechanism of action of SCH 16 in relation to JEV replication

To understand the possible mechanism of action in relation to the replicative cycle of JEV, the compounds that showed 100% inhibition of viral plaques were evaluated by *in vitro *experiments detailed below.

#### Determining kinetics of JEV replication in PS cells

A 24 well plate containing sterile cover slips in each well was seeded with 4 × 10^4 ^cells/well and incubated at 37°C overnight. When the cells were a confluent monolayer, they were infected with JEV (MOI = 1) for 1 hour at 37°C. The monolayer was rinsed thoroughly with sterile PBS and replenished with medium containing 1% FCS. This time point was considered as '0' hour post-infection. Subsequently at 2, 4, 6, 8, 10, 12, 14, 16 and 24 hours post-infection, the medium was harvested to determine the amount of extracellular virus released into the supernatant. At each time point, the cover slip containing cells was also removed, fixed in chilled acetone and stained by Immunofluorescent Assay (IFA) using a monoclonal antibody to envelope protein of JEV to detect the cell bound antigen [38].

#### Understanding the kinetics of the antiviral activity of SCH 16

A 24 well plate was seeded with 4 × 10^4 ^cells/well and incubated at 37°C overnight. To this monolayer JEV was added (MOI = 1) and incubated for 1 hour at 37°C. At the end of adsorption, the virus was removed, the monolayer was rinsed 3 – 4 times using sterile PBS and replenished with MEM containing 1% FCS. This time point was considered as 0 hour post-infection. Starting from 0 hour time point, 76 ug/ml (IC_50_) of the compound was added at 2, 4, 6, 8, 10, 12, 14, 16, and 24 hours post-infection and incubated at 37°C. The supernatant fluid was harvested from the respective wells at 48 hours post-infection. The fluid was divided into two parts. One part was used to determine the virus yield in the supernatant fluid (TCID_50_/ml) and the second part of the fluid was used to detect the presence of soluble JEV antigen using an antigen capture ELISA described elsewhere [39]. In order to detect cell bound antigen the cover slip cultures were fixed in chilled acetone for 30 minutes at 4°C and stained using monoclonal antibody to JEV (Clone F2C2) and anti-mouse IgG FITC conjugate by indirect IFA as described earlier. The cells in each well were treated with 750 μl of TRIzol (Invitrogen, USA) for RNA extraction and reverse transcription was carried out using cDNA archive kit (Applied Biosystems, USA) as described below.

#### Real Time PCR using Syber Green I chemistry

Detection of viral RNA was carried out by Real Time PCR using Syber Green I chemistry as described by Shu et al [40] with minor modifications. Briefly, a 120 base pair product of the PreM gene of JEV was amplified using the forward primer F1 (gga gcc atg aag ttg tca aat ttc) and reverse primer R1 (ttg ccc gga ccc aac at) based on the prototype standard strain of JEV (P20778) Gen Bank Ac.No.7080251.

A second set of experiments was designed to estimate the minimum time required for the compound to bring about complete inhibition of JEV replication. A 24 well plate was seeded with 4 × 10^4 ^cells/well in quadruplicates and incubated at 37°C overnight. Confluent PS monolayers were infected with JEV (MOI = 1) and adsorbed for 1 hour at 37°C. Following this, the monolayer was rinsed with sterile PBS and replenished with plain medium containing non-toxic concentration of SCH16. Control wells received plain medium. This time point was considered as '0' hour post-infection. Starting from 0 hour time point, medium containing the compound was removed at 0, 4, 8, 12, and 14 hours post-infection and replenished with MEM containing 1% FCS. At the end of 48 hours incubation, the fluid harvested from one of the quadruplicate set of wells, was evaluated for presence of extracellular virus by titration while soluble antigen was detected using an antigen capture ELISA described earlier. Cells in a second set of wells were trypsinised, re-suspended in maintenance medium and subjected to three freeze thaw cycles to release intracellular virus, which was quantitated by titration. Cells from the third set of wells were stained by an IFA to detect cell bound antigen. The cells in the fourth set of wells were treated with 750 μl of TRIzol (Invitrogen, USA) for RNA extraction and reverse transcription was carried out using cDNA archive kit (Applied Biosystems, USA).

#### Effect of SCH 16 on the translation of JEV

In order to understand the probable action of SCH 16 on the events of viral replication, an *in vitro *translation experiment was carried out using commercially available Transcend™ non-radioactive translation detection system and rabbit reticulocyte lysate kit (Promega, USA). A 24 well plate was seeded with PS cells (4 × 10^4^/ml), incubated at 37°C for 18 to 24 hrs and the monolayer formed was adsorbed with JEV (MOI = 1) for 1 hour. The infected monolayer was rinsed with sterile PBS to remove the unbound virus. To one set of JEV infected monolayer cultures, SCH 16 at non-toxic concentration was added at '0' hour and incubated for 4 hours. Medium containing SCH 16 was removed at 4 post-infection and replenished. To a second set of monolayer cultures, SCH 16 at the same concentration was added at 10 hours post adsorption. The plates were further incubated for 48 hours at 37°C. Appropriate virus and cell controls were included in parallel to the test. At the end of incubation, the cells were treated with 750 μl of TRIzol and the viral RNA was extracted as described earlier. The viral RNA thus obtained, was divided in to two equal parts. One part was subjected to RT-PCR using JEV specific primers to confirm the presence of JEV RNA. The second part was subjected to *in vitro *translation carried out using a commercial kit (Promega, USA) and manufacturer's guidelines. Briefly, a 50 μl reaction containing 35 μl of rabbit reticulocyte lysate, 10 μl of nuclease free water, 1 μl of RNasin (40 U/μl), 1 μl of complete amino acid mixture (1 mM), 1 μl of Transcend ™ tRNA and 2 μl of RNA template was set up at 30°C and incubated for 60 minutes. After the completion of translation reaction, 1 ul of the product was subjected to SDS – PAGE, electroblotted on to a PVDF membrane, blocked with skimmed milk powder solution, reacted with JEV specific monoclonal antibody to visualize the bands.

### *In vivo *evaluation of SCH 16 against JEV

#### Evaluation of non-toxic concentration of the compounds in mice

In order to determine the *in vivo *non-toxic concentrations, the compound SCH 16 (100, 200, 400 and 500 mg/kg body weight) were administered either per orally, or intraperitoneally into four different groups of 4 – 5 weeks old Swiss albino mice (n = 4). Two groups of mice served as normal controls that received plain medium per orally or intraperitoneally. All mice were observed for a period of 45 days for loss or gain in weight, and other evidences of toxicity as compared to the untreated normal mice.

#### In vivo evaluation of SCH 16 by intracerebral challenge

To determine the *in vivo *antiviral potential of SCH 16, three groups of mice (4 mice per group) were injected intracerebrally with 30 μl of virus suspension containing 50 LD_50 _of JEV. This was followed by intraperitoneal administration of SCH16 (100 and 200 mg/Kg body weight) into two groups of mice twice daily for 10 days. A third group of mice served as control animals that received virus intracerebrally and plain MEM intraperitoneally. Animals were monitored for the appearance of symptoms for JEV infections such as paralysis and death.

#### In vivo evaluation of SCH 16 by peripheral challenge

The therapeutic potential of SCH 16 was also evaluated using a peripheral challenge model wherein JEV (50LD_50_) was injected (200 μl) intraperitoneally into four groups of 4–5 weeks old mice (n = 4). Two hours later, each group received 30 μl of 1% sterile starch intracerebrally to facilitate virus entry into the brain. This was followed by oral administration of SCH 16 in a dose of 200, 400 or 500 mg/kg body weight twice daily into the three respective groups for 12 days. A fourth group of mice (n = 4) served as 'no drug controls' which received virus intraperitoneally, starch intracerebrally and plain MEM orally while a fifth group of mice (n = 4) served as sham controls and received MEM intraperitoneally and starch intracerebrally. Mice were observed every day for 20 days post-infection for the appearance of symptoms and death. At the end of the observation period the mice that survived the infection were sacrificed, brains harvested and subjected to JEV antigen detection by IFA, JEV nucleic acid detection by real-time PCR and virus isolation using shell vial method [41].

## Competing interests

The authors declare that they have no competing interests.
